# A Toxic Friend: Genotoxic and Mutagenic Activity of the Probiotic Strain Escherichia coli Nissle 1917

**DOI:** 10.1128/mSphere.00624-21

**Published:** 2021-08-11

**Authors:** Jean-Philippe Nougayrède, Camille V. Chagneau, Jean-Paul Motta, Nadège Bossuet-Greif, Marcy Belloy, Frédéric Taieb, Jean-Jacques Gratadoux, Muriel Thomas, Philippe Langella, Eric Oswald

**Affiliations:** a IRSD, INSERM, INRAE, Université de Toulouse, ENVT, Toulouse, France; b Micalis, INRAE, Jouy-en-Josas, France; c CHU Toulouse, Hôpital Purpan, Service de Bactériologie-Hygiène, Toulouse, France; UTMB

**Keywords:** *Escherichia coli*, probiotic, colibactin, genotoxin

## Abstract

The probiotic Escherichia coli strain Nissle 1917 (DSM 6601, Mutaflor), generally considered beneficial and safe, has been used for a century to treat various intestinal diseases. However, Nissle 1917 hosts in its genome the *pks* pathogenicity island that codes for the biosynthesis of the genotoxin colibactin. Colibactin is a potent DNA alkylator, suspected to play a role in colorectal cancer development. We show in this study that Nissle 1917 is functionally capable of producing colibactin and inducing interstrand cross-links in the genomic DNA of epithelial cells exposed to the probiotic. This toxicity was even exacerbated with lower doses of the probiotic, when the exposed cells started to divide again but exhibited aberrant anaphases and increased gene mutation frequency. DNA damage was confirmed *in vivo* in mouse models of intestinal colonization, demonstrating that Nissle 1917 produces the genotoxin in the gut lumen. Although it is possible that daily treatment of adult humans with their microbiota does not produce the same effects, administration of Nissle 1917 as a probiotic or as a chassis to deliver therapeutics might exert long-term adverse effects and thus should be considered in a risk-versus-benefit evaluation.

**IMPORTANCE** Nissle 1917 is sold as a probiotic and considered safe even though it has been known since 2006 that it harbors the genes for colibactin synthesis. Colibactin is a potent genotoxin that is now linked to causative mutations found in human colorectal cancer. Many papers concerning the use of this strain in clinical applications ignore or elude this fact or misleadingly suggest that Nissle 1917 does not induce DNA damage. Here, we demonstrate that Nissle 1917 produces colibactin *in vitro* and *in vivo* and induces mutagenic DNA damage. This is a serious safety concern that must not be ignored in the interests of patients, the general public, health care professionals, and ethical probiotic manufacturers.

## INTRODUCTION

Escherichia coli Nissle 1917 is an intestinal strain originally isolated during the first world war. Nissle 1917 is a potent competitor of different enteropathogens in the gut (1). Consequently, it has been used for a century as a treatment for diarrhea and more recently for other intestinal disorders such as inflammatory bowel diseases (IBDs). The use of Nissle 1917 is recommended for maintaining remission in ulcerative colitis (2, 3). It is used as a probiotic in human medicine in Germany, Australia, Canada, and other countries under the name “Mutaflor.” Nissle 1917 is also a popular chassis to engineer therapeutic bacteria for vaccine, diagnostics, biosensors, and drug development (4). The popularity of Nissle 1917 resides not only in its “natural” beneficial properties but also in the general acceptance that it is harmless and safe. Its safety profile is based in part on the belief that Nissle 1917 does not produce any toxin associated with pathogenic strains of E. coli. Although this statement is still propagated in the recent biomedical literature, it was shown in 2006 that Nissle 1917 hosts a 54-kb *pks* island coding for nonribosomal and polyketide synthases (NRPS and PKS, respectively) allowing synthesis of a hybrid peptide-polyketide metabolite called colibactin (5, 6).

Colibactin is a genotoxin that binds and cross-links the opposite strands of DNA, resulting in DNA damage and gene mutagenesis in eukaryotic cells (5, 7–12). Colibactin is a virulence factor during systemic infection (13–15) and plays a substantial role in colorectal cancer. Indeed, colibactin-producing E. coli promote colorectal cancer in mouse models (16, 17), and the DNA mutational signature of colibactin has been found in cohorts of patients with colorectal cancer, including in the *APC* cancer driver gene (9, 11, 18). A conflicting report claimed that “no genotoxicity is detectable for E. coli strain Nissle 1917 by standard *in vitro* and *in vivo* tests” (19), but the authors used assays that are suboptimal to demonstrate the production and mutagenicity of colibactin, such as the use of Salmonella reporter bacteria that are killed by the microcins produced by Nissle 1917 (20, 21). Recently, in a study using stem cell-derived human intestinal organoids to evaluate the safety of the probiotic, Nissle 1917 “was found to be safe” (22), while exposure of such organoids to *pks*^+^ E. coli induced the colibactin-specific mutational signature (11). Here, we examined the production and genotoxicity of colibactin by Nissle 1917 *in vitro*, using assays adapted to the described mode of action of the toxin, and *in vivo* in two mouse models.

## RESULTS

### Nissle 1917 produces colibactin and induces DNA cross-links in infected epithelial cells.

**FIG 1:**
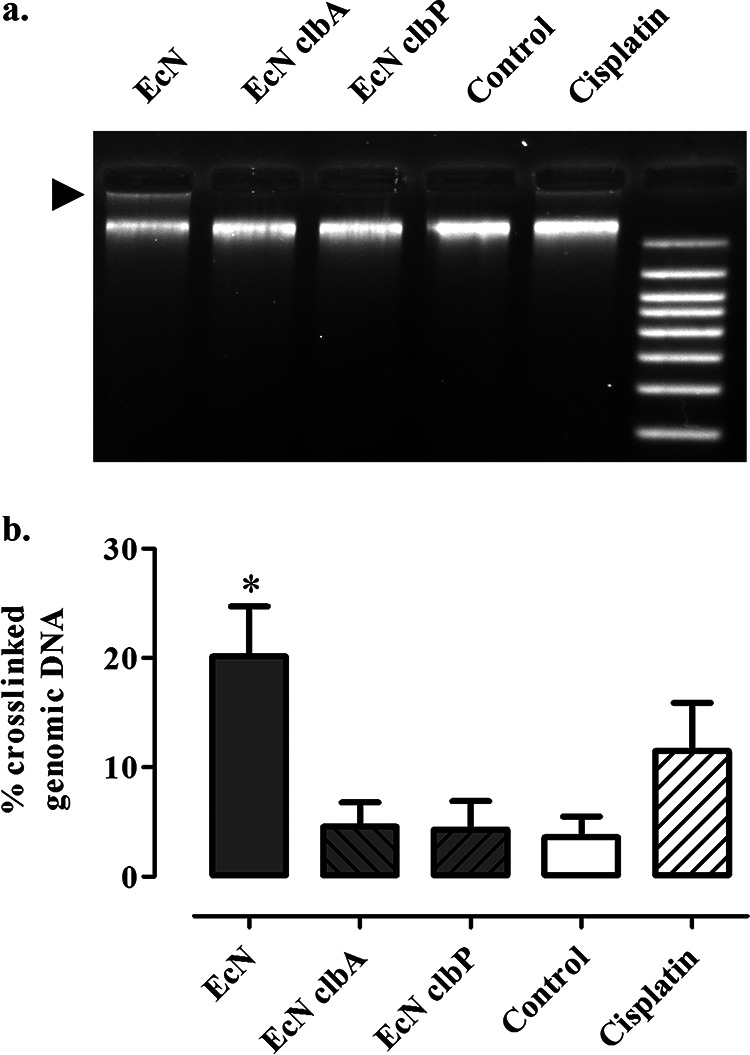
E. coli Nissle 1917 induces interstrand cross-links in the host cell genomic DNA. (a) HeLa cells were infected for 4 h at a multiplicity of infection of 400 bacteria per cell with E. coli Nissle (EcN) or *clbA* or *clbP* isogenic mutants, were left uninfected, or were treated 4 h with 100 μM cisplatin. Then, the cell genomic DNA was purified and analyzed by denaturing electrophoresis. The arrow points to the nonmigrating DNA that remained in the loading well. (b) The DNA signal in the top nonmigrating band relative to the total DNA signal in the lane was determined by image analysis in ImageJ. The mean percentages of cross-linked DNA and standard errors of the means (*n* = 3 independent experiments) are shown. *, *P* < 0.05 compared to control, one-way analysis of variance (ANOVA) with Dunnett posttest.

**FIG 2:**
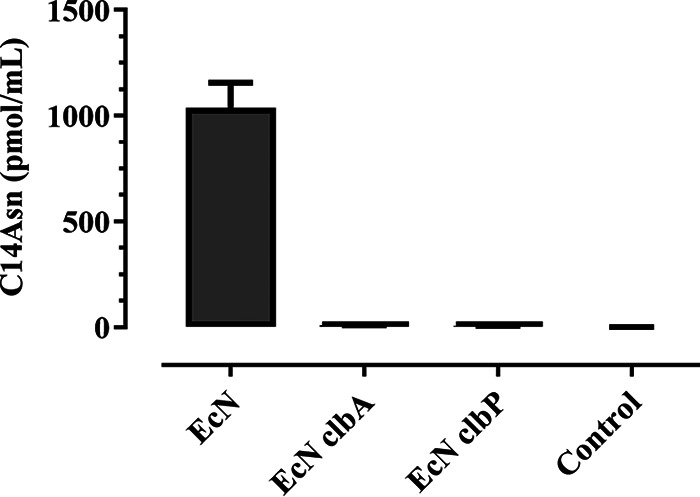
Production of colibactin cleavage product C14-Asn by Nissle 1917 during infection of HeLa cells. The cells were infected as described for Fig. 1 with E. coli Nissle (EcN) or *clbA* or *clbP* mutants or were left uninfected, and then the coculture supernatant was collected and the C14-Asn cleavage product was quantified by liquid chromatography-mass spectrometry (LC-MS). The means and standard errors of the means (*n* = 3 biological replicates) are shown.

DNA interstrand cross-links generated by colibactin impair the denaturation of DNA and thus inhibit its electrophoretic mobility under denaturing conditions (7). We examined whether infection of epithelial cells with Nissle 1917 could induce cross-links in host genomic DNA. Cultured human epithelial HeLa cells were exposed to live E. coli Nissle 1917 for 4 h, and then the cell genomic DNA was purified and analyzed by denaturing gel electrophoresis. In contrast to the DNA of control cells, which migrated as a high-molecular-weight band, a fraction of the DNA of the cells exposed to Nissle 1917 remained in the loading well (Fig. 1). Similar genomic DNA with impaired electrophoretic migration was observed in cells treated with cisplatin, a DNA cross-linking agent (Fig. 1a). Crosslinked genome DNA was also observed in human colorectal cancer HT-29 cells and in nontransformed rat epithelial intestinal IEC-6 cells exposed to Nissle 1917 (see Fig. S1 in the supplemental material). In contrast, a Nissle 1917 mutant for the phosphopantetheinyl transferase ClbA, required for activation of the NRPS and PKS in the *pks* pathway (14), did not induce nonmigrating genomic DNA (Fig. 1a and b). Similarly, no cross-linking activity was detected with the Nissle 1917 strain mutated for the peptidase ClbP that cleaves the inactive precolibactin to release the cleavage product C14-Asn and generate the mature active colibactin (23) (Fig. 1a and b; Fig. S1). Mature colibactin is not detectable directly, but its stable cleavage product can be detected by mass spectrometry. Using this technique, C14-Asn was readily detected in the cell infection medium of the wild-type Nissle 1917 but not in that of the *clbP* mutant (Fig. 2). We also observed the DNA interstrand cross-links in exogenous DNA exposed to the wild-type Nissle 1917 but not to the *clbA* and *clbP* mutants (see Fig. S2). Together, these results demonstrate that Nissle 1917 synthesizes mature DNA-cross-linking colibactin.

10.1128/mSphere.00624-21.1 FIG S1 E. coli Nissle 1917 induces interstrand cross-links in the genome DNA of human colorectal cancer HT-29 cells and nontumor rat intestinal crypt IEC-6 cells. The cells were infected for 4 h at a multiplicity of infection of 400 bacteria per cell with E. coli Nissle (EcN) or the *clbP* isogenic mutant, were left uninfected, or were treated 4 h with 100 μM cisplatin. Then, the cell genomic DNA was purified and analyzed by denaturing electrophoresis. The arrows point to the nonmigrating DNA that remained in the loading well. Download FIG S1, TIF file, 0.8 MB. Copyright © 2021 Nougayrède et al. 2021 Nougayrède et al. https://creativecommons.org/licenses/by/4.0/ This content is distributed under the terms of the Creative Commons Attribution 4.0 International license.

10.1128/mSphere.00624-21.2 FIG S2 E. coli Nissle 1917 induces interstrand cross-links in exogenous DNA. (a) Linearized plasmid double-strand DNA was incubated 40 min with E. coli Nissle (EcN) (inoculum of 0.75, 1.5, 3, or 6 × 10^6^ bacteria in 100 μl, grown 3.5 h) or with the *clbA* or *clbP* mutants (6 × 10^6^ bacteria in 100 μl) or treated 4 h with cisplatin and then analyzed by denaturing gel electrophoresis. (b) Quantification of panel a; the percentages of the DNA signal in the upper cross-linked band relative to the total DNA signal in the lane was determined by image analysis. Download FIG S2, TIF file, 0.8 MB. Copyright © 2021 Nougayrède et al. 2021 Nougayrède et al. https://creativecommons.org/licenses/by/4.0/ This content is distributed under the terms of the Creative Commons Attribution 4.0 International license.

### Infection with Nissle 1917 induces the recruitment of the DNA repair machinery.

**FIG 3:**
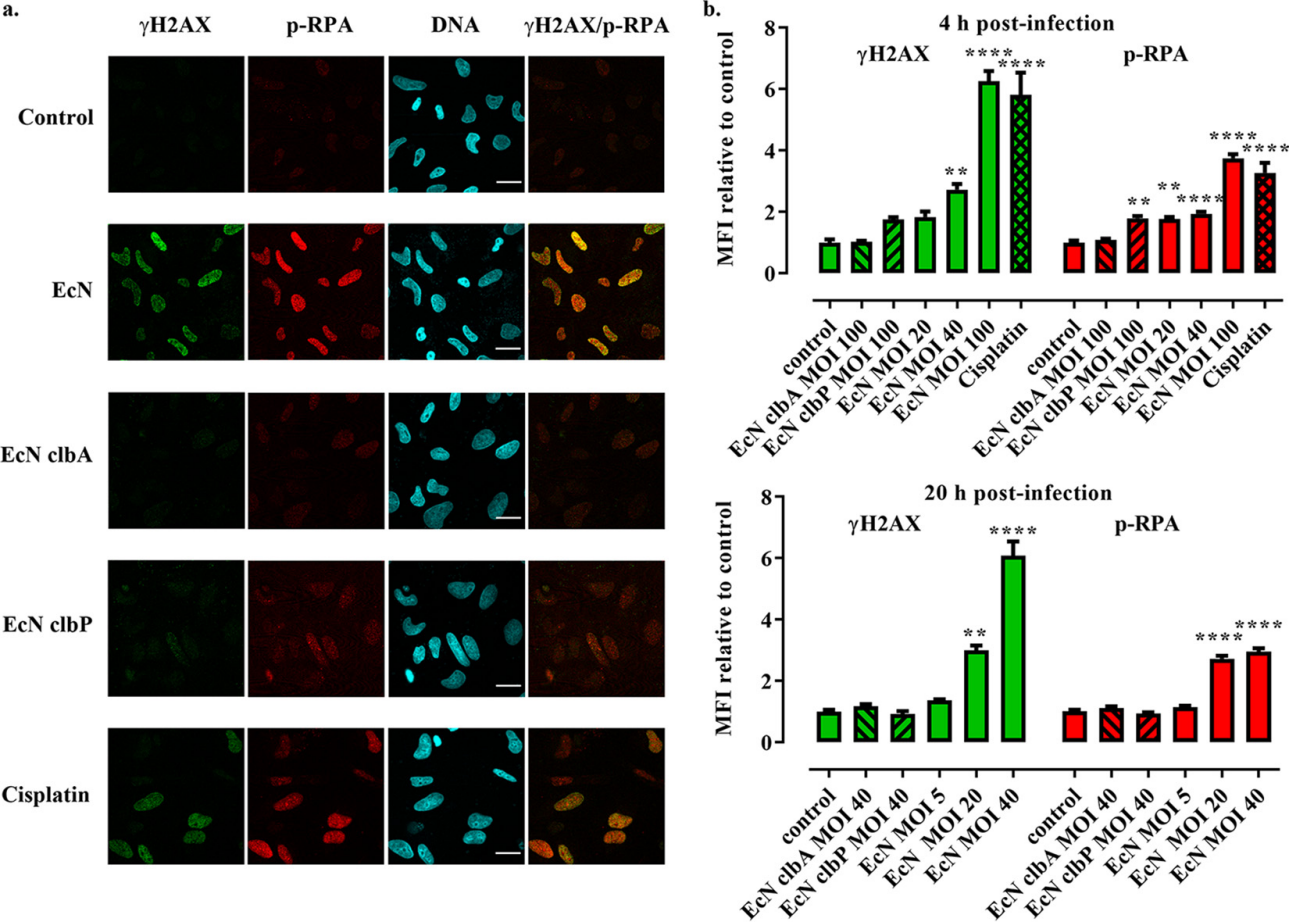
Formation of phosphorylated RPA and H2AX nuclear repair foci in HeLa cells infected with E. coli Nissle 1917. (a) HeLa cells were exposed 4 h to E. coli Nissle 1917 (EcN) or the *clbA* or *clbP* mutants (MOI of 100) or treated with cisplatin and then immunostained for phosphorylated H2AX (γH2AX) and phosphorylated RPA (p-RPA) 4 h later. DNA was counterstained with DAPI. Bars, 20 μm. (b) Cells were infected at the given MOI and immunostained at 4 and 20 h after infection. The mean fluorescence intensities (MFIs) of γH2AX and p-RPA within the nuclei, relative to that in control uninfected cells, were determined by image analysis using a macro in ImageJ. The means and standard errors, measured in at least 70 nuclei for each group, are shown. **, *P* < 0.01, ****, *P* < 0.0001 (one-way ANOVA with Dunnett posttest, compared to control).

It was recently shown that upon formation of DNA cross-links by colibactin, the cells recruit the kinase ataxia telangiectasia and Rad3-related (ATR), which phosphorylate Ser33 of the replication protein A-32 (RPA) in nuclear DNA repair foci together with phosphorylated histone γH2AX (7). Immunofluorescence of Ser33-phosphorylated RPA (p-RPA) and γH2AX showed nuclear foci of both markers in HeLa cells 4 h after infection with Nissle 1917 or following treatment with the cross-linking drug cisplatin but not after infection with the *clbA* or *clbP* mutants (Fig. 3a). The γH2AX and p-RPA foci increased with the multiplicity of infection (MOI) of wild-type Nissle 1917 and remained plainly measurable 20 h after infection, even at the low MOI of 20 bacteria per cell (Fig. 3b). The γH2AX and p-RPA foci were also observed in HT-29 and IEC-6 cells infected with Nissle 1917 but not in the *clbP* mutant (see Fig. S3). Together, these results demonstrate that Nissle 1917 induces dose- and time-dependent DNA cross-links in exposed cells, resulting in cognate DNA repair machinery recruitment.

10.1128/mSphere.00624-21.3 FIG S3 Formation of phosphorylated RPA and H2AX nuclear repair foci in IEC-6 cells (a) and HT-29 cells (b) infected with E. coli Nissle 1917. The cells were exposed (MOI = 40) 4 h to E. coli Nissle (EcN WT) or the *clbP* mutant and then immunostained for phosphorylated H2AX (γH2AX) and phosphorylated RPA (p-RPA) 20 h later. DNA was counterstained with DAPI. Bars, 25 μm. The mean fluorescence intensities (MFIs) of γH2AX and p-RPA within the nuclei relative to that in control cells were determined by image analysis. The means and standard errors, measured in at least 69 nuclei for each group, are shown. ****, *P* < 0.0001 (one-way ANOVA with Dunnett posttest, compared to control). Download FIG S3, TIF file, 1.3 MB. Copyright © 2021 Nougayrède et al. 2021 Nougayrède et al. https://creativecommons.org/licenses/by/4.0/ This content is distributed under the terms of the Creative Commons Attribution 4.0 International license.

### Exposure to low numbers of Nissle 1917 induces abnormal mitosis and increased gene mutation frequency.

**FIG 4:**
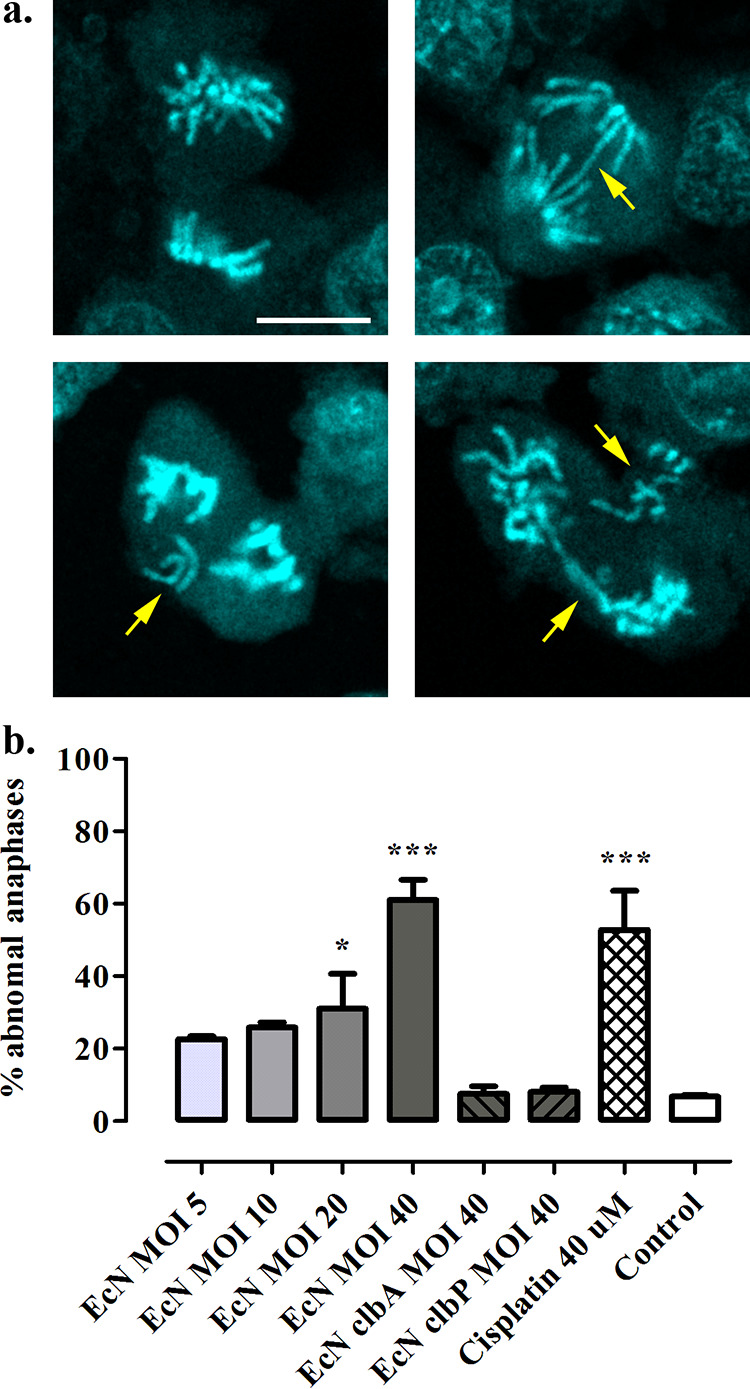
Infection with E. coli Nissle induces aberrant anaphase. (a) Anaphase bridges, lagging chromosomes, and multipolar mitosis (arrows) in CHO cells 20 h following infection with E. coli Nissle. DNA was stained with DAPI and observed by confocal microscopy. Bar, 20 μm. (b) Aberrant anaphase index in CHO cells 20 h following infection with EcN at the given MOI or with the *clbA* and *clbP* mutants or following treatment with cisplatin. The means and standard errors, measured in three independent experiments, are shown. *, *P* < 0.05; ***, *P* < 0.001 (one-way ANOVA with Dunnett posttest compared to control).

Infection with colibactin-producing E. coli at low MOI can lead to incomplete DNA repair in a subset of the cell population, allowing cell division to restart, the formation of aberrant anaphases, and, ultimately, increased gene mutation frequency (8). We thus tested whether infection with Nissle 1917 induced these phenotypes in epithelial CHO cells that have stable chromosomes and are amenable to gene mutation assay. CHO cells exposed to low numbers of wild-type Nissle 1917 showed abnormal mitotic figures 20 h after infection (Fig. 4a). We observed lagging chromosomes, multipolar mitosis, and anaphase DNA bridges in cells infected with Nissle 1917 or treated with cisplatin (Fig. 4a). The abnormal mitotic index increased with the MOI of the wild-type Nissle 1917 strain, whereas it remained at background level in cells exposed to the highest MOI of the *clbA* or *clbP* mutants (Fig. 4b).

Mitotic errors can lead to an accumulation of DNA damage, which in turn favors gene mutations (24, 25). We thus next assessed gene mutation frequencies at the hypoxanthine-guanine phosphoribosyltransferase (*hprt*) loci after infection of CHO cells (Table 1). We found a 2-fold increase in 6-thioguanine-resistant (*hprt* mutant) colonies after infection with wild-type Nissle 1917 at an MOI of 10 compared with uninfected cells or cells that were infected with the *clbA* or *clbP* mutant. The mutation frequency was similar to that previously observed with a laboratory E. coli strain hosting the *pks* island at the same MOI (8) but did not reach statistical significance. Infection with Nissle 1917 at an MOI of 20 resulted in a significant increase of *hprt* mutation frequency. Treatment with cisplatin also resulted in a significant increase of *hprt* mutants, with a mutation frequency similar to that reported in the literature (26). We conclude that Nissle 1917 is mutagenic.

**TABLE 1:** *hprt* mutant frequencies following infection with E. coli Nissle 1917

Treatment^*a*^	MF (×10^−6^)^*b*^	*P* value^*c*^
Control	5.99 ± 0.98	
Cisplatin 10 μM	25.25 ± 5.83	0.006^*d*^
Cisplatin 15 μM	47.62 ± 12.60	0.002^*d*^
EcN, MOI of 5	5.66 ± 0.71	0.685
EcN, MOI of 10	11.98 ± 5.99	0.425
EcN, MOI of 20	14.49 ± 8.37	0.023^*d*^
EcN *clbA* mutant, MOI of 20	5.46 ± 0.28	0.450
EcN *clbP* mutant, MOI of 20	4.94 ± 0.51	0.168

*^a^*Treatments were 1 h cisplatin or infection with EcN or *clbA* or *clbP* mutants at the given multiplicity of infection (MOI).

*^b^*MF, mutant frequency. The values are the means and standard errors from three independent infection experiments.

*^c^*Statistical analysis compared to control was performed using a two-tailed *t* test on the log-transformed data.

d Significant difference.

### Nissle 1917 induces DNA damage to intestinal cells *in vivo*.

**FIG 5:**
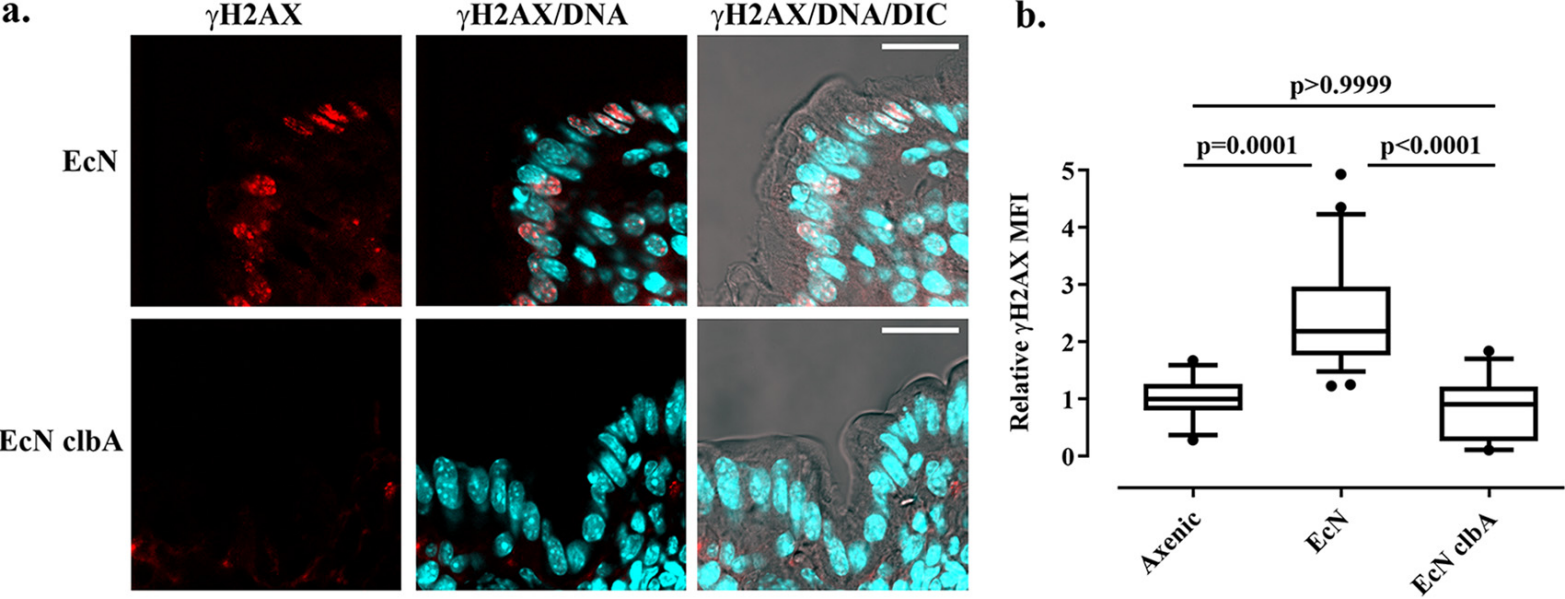
γH2AX foci in gut cells of mice monoassociated with E. coli Nissle 1917. Adult BALB/c mice were monocolonized 7 days with wild-type E. coli Nissle 1917 (EcN) or the *clbA* mutant or were kept axenic. (a) γH2AX in histological sections of the colon was examined by immunofluorescence and confocal microscopy (red). DNA was counterstained with DAPI, and the tissue was visualized by differential interference contrast (DIC). Bars, 10 μm. (b) The mean fluorescence intensities (MFIs) of γH2AX within the nuclei, relative to that measured in the axenic animals, were determined by automated image analysis in ImageJ. The whisker plots show the medians, 10th to 90th percentiles, and outliers measured in at least 20 microscopic fields in 3 axenic and 5 monoassociated animals. The results of a Kruskal-Wallis and Dunn’s multiple-comparison test are shown.

To test whether Nissle 1917 produces colibactin *in vivo* in the gut lumen and induces DNA damage to intestinal cells, we first used a simplified model of intestinal colonization; adult axenic BALB/c mice were inoculated with Nissle 1917 or the *clbA* mutant or with sterile phosphate-buffered saline (PBS). Seven days after inoculation, the mice were sacrificed, and fecal and colon tissue samples were collected. The mice monoassociated with Nissle 1917 or hosting the *clbA* mutant exhibited similar fecal counts of ∼10^9^ CFU/g of feces. We assessed by immunohistology histone γH2AX in the colon. Nuclear γH2AX foci were readily observed in the enterocytes exposed to Nissle 1917 but not in those from animals inoculated with the *clbA* mutant, which exhibited background γH2AX levels similar to that of the axenic controls (Fig. 5a and b).

**FIG 6:**
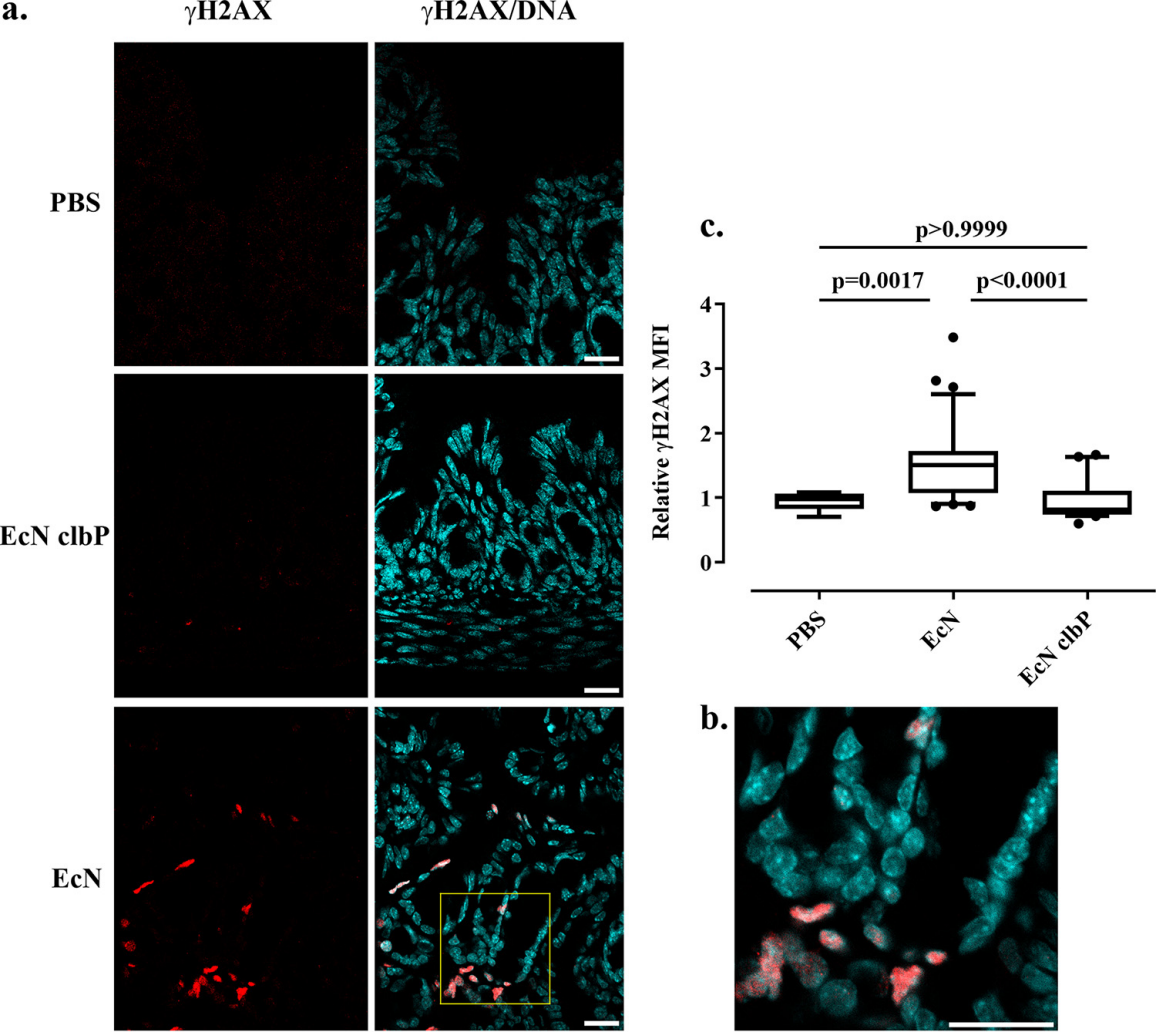
γH2AX foci in gut cells of mouse pups treated by E. coli Nissle 1917 by gavage. (a) Mice pups were given orally approximatively 2.5 × 10^8^ wild-type E. coli Nissle (EcN) or the *clbP* mutant or the PBS vehicle and then sacrificed 6 h later. Phosphorylated H2AX (γH2AX) in histological sections of the intestinal epithelium was examined by immunofluorescence (red) and confocal microscopy. DNA was counterstained with DAPI. Bars, 20 μm. (b) Close-up of the region shown in yellow. Bar, 20 μm. (c) The mean fluorescence intensities (MFIs) of γH2AX within the nuclei, relative to that in the controls, were determined by automated image analysis in ImageJ. The whisker plots show the medians, 10th to 90th percentiles, and outliers measured in at least 10 microscopic fields for each group in 3 controls (PBS) or 5 treated (Nissle 1917 or *clbP* mutant) animals. The results of a Kruskal-Wallis and Dunn’s multiple-comparison test are shown.

Nissle 1917 is used not only in adults but also in infants and toddlers. To further examine production of colibactin *in vivo*, we used a second *in vivo* model in which 8-day-old Swiss mouse pups were given *per os* ∼10^8^ CFU of Nissle 1917 or the *clbP* mutant or PBS. Six hours after inoculation, the colon epithelium was examined for formation of γH2AX foci. Animals treated with Nissle 1917 exhibited significant levels of nuclear γH2AX compared to that in controls treated with PBS (Fig. 6a and b). In contrast, the animals treated with the *clbP* mutant that does not produce colibactin showed background levels of γH2AX (Fig. 6a and b). Together these results indicated that Nissle 1917 induces *in vivo* DNA damage to epithelial cells.

## DISCUSSION

The identification of a colibactin mutation signature in human colorectal cancer tissues (9, 11, 18) and also in colonic crypts from healthy individuals under the age of 10 years (27) proves that colibactin is expressed within the human gut (including in children) and links colibactin exposure to colorectal cancer. Colibactin is now a suspected prooncogenic driver, especially in IBD patients (28). Nissle 1917 has been used as a probiotic for various clinical applications since its isolation more than 100 years ago. It has shown some efficacy to treat IBDs such as Crohn’s disease and ulcerative colitis. In this study, we demonstrate that Nissle 1917 synthesizes colibactin, *in vitro* and *in vivo* in the mouse gut lumen, and inflicts mutagenic DNA damage. Even in low numbers, DNA cross-links are catastrophic damages that obstruct basic DNA processes, since they prevent the strand separation required for polymerase functions. The cross-links notably perturb the replication machinery, resulting in replication stress, accumulation of DNA bound by RPA, and activation of the kinase ATR that in turn phosphorylates RPA and histone variant H2AX (7, 29, 30). We observed that cells exposed to Nissle 1917 at low MOI (hence, numbers of bacteria more relevant to those occurring *in vivo*) entered an error-prone repair pathway, exhibiting mitotic aberrations and increased gene mutation frequency, similar to that observed with other *pks*^+^ E. coli strains (8, 10). Thus, Nissle 1917 is genotoxigenic and mutagenic. This is of concern for patients and participants in clinical trials using Nissle 1917, such as the trial in Finland in which more than 250 young children will be inoculated with this strain (https://clinicaltrials.gov/ct2/show/NCT04608851).

Our results stand in contrast to that reported by Dubbert and colleagues who claimed that Nissle 1917 does not have detectable mutagenic activity using standard tests (19). However, the assays they used cannot detect colibactin-associated mutagenic damage. Indeed, to examine whether Nissle 1917 could induce mutagenic DNA damage, Dubbert et al. (19) used an Ames test in which Salmonella enterica serovar Typhimurium reporter bacteria were exposed to Nissle 1917, and then Salmonella growth was expected upon mutagenesis. However, Salmonella bacteria are readily killed by the siderophores/microcins produced by Nissle 1917 (20, 21); thus, the absence of growth of the reporter bacteria was incorrectly interpreted as an absence of effect of colibactin. In addition, Dubbert et al. (19) used a standard comet assay that can detect a variety of DNA lesions through electrophoresis of broken DNA but which cannot detect DNA cross-links that inhibit DNA electrophoretic mobility (7, 12, 31). Thus, the standard assays used by Dubbert et al. (19) were inappropriate, in contrast to the assays used in the present and other works (12, 32), to highlight the DNA-damaging activity and genotoxicity of colibactin produced by Nissle 1917. Along the same line, Pradhan and Weiss recently reported that human epithelial intestinal organoids infected with Nissle 1917 did not exhibit adverse phenotypes (such as loss of barrier function or apoptotic cell death) and thus concluded that the probiotic was safe (22). However, DNA damage triggers a complex interplay between DNA repair, cell death, and survival (33). As a result, DNA-damaging and mutagenic activities are not outwardly apparent in the form of cell senescence or death but require careful investigation with appropriate assays. This is exemplified in this work where Nissle 1917-infected cells survived and pursued division during 21 days before examining gene mutation.

We demonstrate, using two mouse models, that Nissle 1917 synthesizes colibactin in the mouse gut and induces DNA damage in colon cells. A limitation of the present study is that we did not examine whether this DNA damage could promote colorectal cancer; thus, the tumorigenesis potential of Nissle 1917 remains to be tested using colorectal cancer or IBD mouse models. In addition, mouse models do not fully recapitulate the human intestine, in particular, its complex microbiota, epithelial, and intestinal barrier functions. However, in human patients, Nissle 1917 is typically used in the context of IBDs, where the gut is inflamed, the intestinal barrier is dysfunctional, and the microbiota is dysbiotic. Importantly, intestinal inflammation was shown to upregulate *pks* genes (28, 34, 35). Inflammation and dysbiosis are also known to allow the expansion of the E. coli population, including that of Nissle 1917, alongside the epithelium (36–38). Moreover, Nissle 1917 is typically administered in very high numbers (2.5 × 10^9^ to 25 × 10^9^ bacteria in adults, 10^8^ in infants), repeatedly (1 to 4 times daily), for weeks or even longer in the case of ulcerative colitis. Nissle 1917 has been reported to persist in the human gut for months after inoculation (39). Thus, patients treated with this probiotic can be exposed chronically to high numbers of colibactin-producing bacteria, especially in an inflamed context that favors colibactin production, and consequently could be exposed to high levels of mutagenic colibactin. These conditions were shown to promote colon tumorigenesis in colorectal cancer (16).

Nissle 1917 has been used for decades to treat gastrointestinal disorders such as diarrhea and inflammatory bowel diseases, in particular, ulcerative colitis. A large body of literature demonstrates its beneficial effects. For example, its efficacy versus placebo has been shown in infants and toddlers with diarrhea (40). Randomized clinical trials and meta-analyses support the beneficial role of Nissle 1917 in the therapy of ulcerative colitis (41). Nissle 1917 is also an increasingly popular choice to engineer live biotherapeutics (i.e., bacteria genetically designed to treat or prevent a disease) (42). For example, Nissle 1917 has been used successfully as a chassis to deliver an antibiofilm enzyme against Pseudomonas aeruginosa (43) or a microcin induced upon sensing of Salmonella infection (44). Engineered strains of Nissle 1917 have also been constructed to treat obesity through production of *N*-acylphosphatidylethanolamine (45) or to express a phenylalanine-metabolizing enzyme in response to the anoxic conditions in the gut to treat phenylketonuria (46). Considering the widespread use of Nissle 1917 as an efficient probiotic and as a platform to develop live bacterial therapeutics, ensuring its safety is of paramount importance. Genotoxic carcinogens are classically conceived to represent a risk factor with no threshold dose, because little numbers or even one DNA lesion may result in mutation and increased tumor risk (47). Production of mutagenic colibactin by Nissle 1917 is thus a serious health concern that must be addressed.

## MATERIALS AND METHODS

### E. coli EcN strain, mutants, and culture.

The E. coli strain Nissle 1917 used in this study was obtained from Ulrich Dobrindt (University of Münster). The *clbA* and *clbP* isogenic mutants were described previously (48, 49). Before infection, the bacteria were grown overnight at 37°C with 240-rpm agitation in 5 ml of Lennox L broth (LB; Invitrogen) and then diluted 1/20 in prewarmed Dulbecco’s modified Eagle medium (DMEM) with 25 mM HEPES (Invitrogen) and incubated at 37°C with 240-rpm agitation to reach exponential phase (optical density at 600 nm [OD_600_] of 0.4 to 0.5).

### *In vitro* DNA cross-linking assay.

Briefly, 3 × 10^6^ bacteria (or numbers given in the text) were inoculated in 100 μl of DMEM with 25 mM HEPES and incubated at 37°C for 3.5 h, and then EDTA (1 mM) and 400 ng of linearized (BamHI) pUC19 DNA were added and further incubated 40 min. As controls, DNA was left untreated or treated with 100 or 200 μM cisplatin (Sigma). Following centrifugation to pellet the bacteria, the DNA was purified using a Qiagen PCR DNA purification kit before analysis by denaturing gel electrophoresis.

### Denaturing gel DNA electrophoresis.

One-percent agarose gels prepared in a 100 mM NaCl and 2 mM EDTA (pH 8) solution were soaked for 16 h in 40 mM NaOH and 1 mM EDTA electrophoresis running buffer. DNA electrophoresis was performed at room temperature for 45 min at 1 V/cm and then 2 h at 2 V/cm. Following neutralization by serial washes in 150 mM NaCl-100 mM Tris (pH 7.4), DNA was stained with GelRed (Biotium) and photographed with flat-field correction and avoiding charge-coupled device (CCD) pixel saturation in a Bio-Rad ChemiDoc XRS system. Images were analyzed using NIH ImageJ: the background was subtracted (100 pixels, rolling ball) and then the lane profiles were plotted and the areas of DNA peaks were measured.

### Cell culture and infection.

HeLa and CHO cells were cultivated in a 37°C 5% CO_2_ incubator and maintained by serial passage in DMEM GlutaMAX and MEMα (Invitrogen), respectively, both supplemented with 10% fetal calf serum (FCS), 50 μg/ml gentamicin, and 1% nonessential amino acids (Invitrogen). Briefly, 3 × 10^5^ cells/well were seeded in 6-well plates (TPP) or 3.5 × 10^4^ cells/well in 8-chambers slides (Falcon) and grown 24 h. Cells were washed 3 times in Hanks’ balanced salt solution (HBSS; Invitrogen) before infection in DMEM with 25 mM HEPES at a given multiplicity of infection (MOI; number of bacteria per cell at the onset of infection). Following the 4 h coculture, the cells were washed 3 times with HBSS and then incubated in complete cell culture medium supplemented with 200 μg/ml gentamicin for the specified times (0, 4, or 20 h) before analysis.

### Extraction and quantification of C14-Asn.

After HeLa cell infection in 6-wells plates, 1 ml of the cell supernatant containing the bacteria was collected, the samples were lysed by bead beating, the lipids were separated by solid-phase extraction, and C14-Asn was quantified by high-performance liquid chromatography coupled to mass spectrometry at the MetaToul Lipidomics facility (Inserm UMR1048, Toulouse, France), as previously described (50).

### *In cellulo* genomic DNA cross-linking assay.

The cells were infected 4 h or treated 4 h with 100 μM cisplatin (Sigma) and then collected immediately by trypsinization. The cell genomic DNA was purified with a Qiagen DNeasy blood and tissue kit and analyzed by denaturing gel electrophoresis.

### Abnormal anaphase scoring.

Abnormal anaphase quantification was performed as described previously (51). Briefly, 3 h after the end of infection, the cells were trapped in premetaphase by treatment with 0.6 μg/ml nocodazole and released for 55 min without nocodazole to reach anaphase. The slides were fixed, stained with 4′,6-diamidino-2-phenylindole (DAPI) and examined by confocal microscopy as described below. The anaphases were scored in three independent experiments.

### Gene mutation assay.

CHO cells were treated 4 days with culture medium supplemented with 10 mM deoxycytidine, 200 mM hypoxanthine, 0.2 mM aminoprotein, and 17.5 mM thymidine (Sigma) to eliminate preexisting *hprt* mutants. CHO cells were infected 4 h with Nissle 1917 or *clbA* or *clbP* mutants or were treated with cisplatin and then washed and cultured 1 week in normal cell culture medium and passaged in 10-cm dishes seeded with 3 × 10^5^ cells using culture medium supplemented with 30 μM 6-thioguanine (6-TG; Sigma). Cells were also plated without 6-TG to determine plating efficiency. The culture medium was changed twice a week for 21 days. Then, plates were fixed with 4% formaldehyde and stained with methylene blue.

### Animal studies.

All procedures were carried out according to European and French guidelines for the care and use of laboratory animals. The experimentations were approved by Regional Council of Ethics for animal experimentation. Specific-pathogen-free (SPF) pregnant Swiss mice obtained from Janvier (Le Genest, St Isle, France) were housed under SPF conditions in the Inserm Purpan animal facility (Toulouse, France). Eight-day-old mice pups received *per os* a drop (approximately 25 μl) of bacteria suspended (10^10^ CFU/ml) in PBS and were sacrificed 6 h later (protocols 16-U1220-JPN/FT-010 and 17-U1220-EO/PM-461). Germ-free BALB/c mice were housed in the breeding facility of ANAXEM (INRAE, UMR1319 MICALIS, Jouy-en-Josas, France). Axenic animals were inoculated once by intragastric gavage with 10^8^ bacteria suspended in PBS and sacrificed 7 days later (protocol APAFIS number 3441-2016010614307552 v1). Colon tissue samples were fixed 24 h in neutral buffered formalin, dehydrated in ethanol, and embedded in paraffin.

### γH2AX and p-RPA immunofluorescence analysis.

Four or 20 h after infection, HeLa cells were pre-extracted 5 min in PBS with 0.1% Triton X-100 before a 30-min fixation in PBS with 4% formaldehyde. Following permeabilization in 0.1% Triton X-100 and blocking in MAXblock medium (Active Motif), the cells were stained 3 h with antibodies against γH2AX (1:500, JBW301; Millipore) and S33p-RPA32 (1:500, A300-264A; Bethyl) diluted in MAXblock-0.05% Triton X-100. The cells were washed 3 times in PBS-0.05% Triton X-100 and incubated 1 h with anti-mouse Alexa Fluor 488 and anti-rabbit Alexa Fluor 568 antibodies (Invitrogen) diluted 1:500 in MAXblock medium with 1 μg/ml DAPI (Sigma). The cells were washed again, mounted in Fluoroshield medium (Sigma), and examined with a Zeiss LSM 710 or Leica SP8 laser scanning confocal microscope in sequential mode. The mean fluorescence intensities (MFIs) of γH2AX and p-RPA within the nuclei were analyzed using an NIH ImageJ macro: the nuclei were identified in the DNA image (following a 0.5-μm Gaussian blur and default autothreshold) and copied in the ROI manager to measure their corresponding MFIs in the green and red channels.

For immunohistological staining of γH2AX in intestinal tissues, sections (5 or 8 μm) were deparaffinized by serial washes in xylene and ethanol and then rehydrated with water. The antigens were unmasked in HBSS-0.05% trypsin-0.02% EDTA at 37°C for 6 min and then in sodium citrate buffer (10 mM sodium citrate, 0.05% Tween 20, pH 6.0) for 30 min at 80 to 95°C. Following a 1-h cooling to room temperature and blocking 1 h in 0.3% Triton X-100-MAXblock medium, the tissues were stained 16 h at 4°C with primary antibodies against γH2AX (1:200, 20E3; Cell Signaling Technology) diluted in the blocking medium. The slides were washed 3 times in PBS-0.05% Triton X-100 and incubated 1 h with anti-rabbit Alexa Fluor 568 antibody diluted 1:200 in MAXblock medium with 1 μg/ml DAPI. The slides were washed again, mounted, and examined as described above.

### Statistical analyses.

Statistical analyses were performed using GraphPad Prism 9. Analysis of mutant frequencies was performed using a two-tailed *t* test on the log-transformed data to ensure data normality and to correct variance heterogeneity (26).

## References

[1] Nissle A. 1959. [On coli antagonism, dysbacteria and coli therapy]. Med Monatsschr 13:489–491. (In German.) 14427355

[2] Floch MH, Walker WA, Madsen K, Sanders ME, Macfarlane GT, Flint HJ, Dieleman LA, Ringel Y, Guandalini S, Kelly CP, Brandt LJ. 2011. Recommendations for probiotic use-2011 update. J Clin Gastroenterol 45 Suppl:S168–S171. doi:10.1097/MCG.0b013e318230928b. 21992958

[3] Kruis W, Frič P, Pokrotnieks J, Lukáš M, Fixa B, Kaščák M, Kamm MA, Weismueller J, Beglinger C, Stolte M, Wolff C, Schulze J. 2004. Maintaining remission of ulcerative colitis with the probiotic *Escherichia coli* Nissle 1917 is as effective as with standard mesalazine. Gut 53:1617–1623. doi:10.1136/gut.2003.037747. 15479682 PMC1774300

[4] Ou B, Yang Y, Tham WL, Chen L, Guo J, Zhu G. 2016. Genetic engineering of probiotic *Escherichia coli* Nissle 1917 for clinical application. Appl Microbiol Biotechnol 100:8693–8699. doi:10.1007/s00253-016-7829-5. 27640192

[5] Nougayrede J-P, Homburg S, Taieb F, Boury M, Brzuszkiewicz E, Gottschalk G, Buchrieser C, Hacker J, Dobrindt U, Oswald E. 2006. *Escherichia coli* induces DNA double-strand breaks in eukaryotic cells. Science 313:848–851. doi:10.1126/science.1127059. 16902142

[6] Homburg S, Oswald E, Hacker J, Dobrindt U. 2007. Expression analysis of the colibactin gene cluster coding for a novel polyketide in *Escherichia coli*. FEMS Microbiol Lett 275:255–262. doi:10.1111/j.1574-6968.2007.00889.x. 17714479

[7] Bossuet-Greif N, Vignard J, Taieb F, Mirey G, Dubois D, Petit C, Oswald E, Nougayrède J-P. 2018. The colibactin genotoxin generates DNA interstrand cross-links in infected cells. mBio 9:e02393-17. doi:10.1128/mBio.02393-17. 29559578 PMC5874909

[8] Cuevas-Ramos G, Petit CR, Marcq I, Boury M, Oswald E, Nougayrède J-P. 2010. *Escherichia coli* induces DNA damage *in vivo* and triggers genomic instability in mammalian cells. Proc Natl Acad Sci USA 107:11537–11542. doi:10.1073/pnas.1001261107. 20534522 PMC2895108

[9] Dziubańska-Kusibab PJ, Berger H, Battistini F, Bouwman BAM, Iftekhar A, Katainen R, Cajuso T, Crosetto N, Orozco M, Aaltonen LA, Meyer TF. 2020. Colibactin DNA-damage signature indicates mutational impact in colorectal cancer. Nat Med 26:1063–1069. doi:10.1038/s41591-020-0908-2. 32483361

[10] Iftekhar A, Berger H, Bouznad N, Heuberger J, Boccellato F, Dobrindt U, Hermeking H, Sigal M, Meyer TF. 2021. Genomic aberrations after short-term exposure to colibactin-producing *E. coli* transform primary colon epithelial cells. Nat Commun 12:1003. doi:10.1038/s41467-021-21162-y. 33579932 PMC7881031

[11] Pleguezuelos-Manzano C, Puschhof J, Huber AR, van Hoeck A, Wood HM, Nomburg J, Gurjao C, Manders F, Dalmasso G, Stege PB, Paganelli FL, Geurts MH, Beumer J, Mizutani T, Miao Y, van der Linden R, van der Elst S, Genomics England Research Consortium, Garcia KC, Top J, Willems RJL, Giannakis M, Bonnet R, Quirke P, Meyerson M, Cuppen E, van Boxtel R, Clevers H. 2020. Mutational signature in colorectal cancer caused by genotoxic *pks*^+^ *E. coli*. Nature 580:269–273. doi:10.1038/s41586-020-2080-8. 32106218 PMC8142898

[12] Wilson MR, Jiang Y, Villalta PW, Stornetta A, Boudreau PD, Carrá A, Brennan CA, Chun E, Ngo L, Samson LD, Engelward BP, Garrett WS, Balbo S, Balskus EP. 2019. The human gut bacterial genotoxin colibactin alkylates DNA. Science 363:eaar7785. doi:10.1126/science.aar7785. 30765538 PMC6407708

[13] Marcq I, Martin P, Payros D, Cuevas-Ramos G, Boury M, Watrin C, Nougayrède J-P, Olier M, Oswald E. 2014. The genotoxin colibactin exacerbates lymphopenia and decreases survival rate in mice infected with septicemic *Escherichia coli*. J Infect Dis 210:285–294. doi:10.1093/infdis/jiu071. 24489107

[14] Martin P, Marcq I, Magistro G, Penary M, Garcie C, Payros D, Boury M, Olier M, Nougayrède J-P, Audebert M, Chalut C, Schubert S, Oswald E. 2013. Interplay between siderophores and colibactin genotoxin biosynthetic pathways in *Escherichia coli*. PLoS Pathog 9:e1003437. doi:10.1371/journal.ppat.1003437. 23853582 PMC3708854

[15] McCarthy AJ, Martin P, Cloup E, Stabler RA, Oswald E, Taylor PW. 2015. The genotoxin colibactin is a determinant of virulence in *Escherichia coli* K1 experimental neonatal systemic infection. Infect Immun 83:3704–3711. doi:10.1128/IAI.00716-15. 26150540 PMC4534652

[16] Arthur JC, Perez-Chanona E, Mühlbauer M, Tomkovich S, Uronis JM, Fan T-J, Campbell BJ, Abujamel T, Dogan B, Rogers AB, Rhodes JM, Stintzi A, Simpson KW, Hansen JJ, Keku TO, Fodor AA, Jobin C. 2012. Intestinal inflammation targets cancer-inducing activity of the microbiota. Science 338:120–123. doi:10.1126/science.1224820. 22903521 PMC3645302

[17] Cougnoux A, Dalmasso G, Martinez R, Buc E, Delmas J, Gibold L, Sauvanet P, Darcha C, Déchelotte P, Bonnet M, Pezet D, Wodrich H, Darfeuille-Michaud A, Bonnet R. 2014. Bacterial genotoxin colibactin promotes colon tumour growth by inducing a senescence-associated secretory phenotype. Gut 63:1932–1942. doi:10.1136/gutjnl-2013-305257. 24658599

[18] Terlouw D, Suerink M, Boot A, van WT, Nielsen M, Morreau H. 2020. Recurrent *APC* splice variant c.835-8A>G in patients with unexplained colorectal polyposis fulfilling the colibactin mutational signature. Gastroenterology 159:1612.e5–1614.e5. doi:10.1053/j.gastro.2020.06.055. 32603656

[19] Dubbert S, Klinkert B, Schimiczek M, Wassenaar TM, von Bünau R. 2020. No genotoxicity is detectable for *Escherichia coli* strain Nissle 1917 by standard *in vitro* and *in vivo* tests. Eur J Microbiol Immunol (Bp) 10:11–19. doi:10.1556/1886.2019.00025. 32363034 PMC7182118

[20] Massip C, Branchu P, Bossuet-Greif N, Chagneau CV, Gaillard D, Martin P, Boury M, Sécher T, Dubois D, Nougayrède J-P, Oswald E. 2019. Deciphering the interplay between the genotoxic and probiotic activities of *Escherichia coli* Nissle 1917. PLoS Pathog 15:e1008029. doi:10.1371/journal.ppat.1008029. 31545853 PMC6776366

[21] Sassone-Corsi M, Nuccio S-P, Liu H, Hernandez D, Vu CT, Takahashi AA, Edwards RA, Raffatellu M. 2016. Microcins mediate competition among Enterobacteriaceae in the inflamed gut. Nature 540:280–283. doi:10.1038/nature20557. 27798599 PMC5145735

[22] Pradhan S, Weiss AA. 2020. Probiotic properties of *Escherichia coli* Nissle in human intestinal organoids. mBio 11:e01470-20. doi:10.1128/mBio.01470-20. 32636253 PMC7343996

[23] Brotherton CA, Balskus EP. 2013. A prodrug resistance mechanism is involved in colibactin biosynthesis and cytotoxicity. J Am Chem Soc 135:3359–3362. doi:10.1021/ja312154m. 23406518

[24] Chatterjee N, Walker GC. 2017. Mechanisms of DNA damage, repair, and mutagenesis. Environ Mol Mutagen 58:235–263. doi:10.1002/em.22087. 28485537 PMC5474181

[25] Levine MS, Holland AJ. 2018. The impact of mitotic errors on cell proliferation and tumorigenesis. Genes Dev 32:620–638. doi:10.1101/gad.314351.118. 29802124 PMC6004076

[26] Silva MJ, Costa P, Dias A, Valente M, Louro H, Boavida MG. 2005. Comparative analysis of the mutagenic activity of oxaliplatin and cisplatin in the *Hprt* gene of CHO cells. Environ Mol Mutagen 46:104–115. doi:10.1002/em.20138. 15887215

[27] Lee-Six H, Olafsson S, Ellis P, Osborne RJ, Sanders MA, Moore L, Georgakopoulos N, Torrente F, Noorani A, Goddard M, Robinson P, Coorens THH, O'Neill L, Alder C, Wang J, Fitzgerald RC, Zilbauer M, Coleman N, Saeb-Parsy K, Martincorena I, Campbell PJ, Stratton MR. 2019. The landscape of somatic mutation in normal colorectal epithelial cells. Nature 574:532–537. doi:10.1038/s41586-019-1672-7. 31645730

[28] Dubinsky V, Dotan I, Gophna U. 2020. Carriage of colibactin-producing bacteria and colorectal cancer risk. Trends Microbiol 28:874–876. doi:10.1016/j.tim.2020.05.015. 32507544

[29] Maréchal A, Zou L. 2015. RPA-coated single-stranded DNA as a platform for post-translational modifications in the DNA damage response. Cell Res 25:9–23. doi:10.1038/cr.2014.147. 25403473 PMC4650586

[30] Vassin VM, Anantha RW, Sokolova E, Kanner S, Borowiec JA. 2009. Human RPA phosphorylation by ATR stimulates DNA synthesis and prevents ssDNA accumulation during DNA-replication stress. J Cell Sci 122:4070–4080. doi:10.1242/jcs.053702. 19843584 PMC2776501

[31] Merk O, Speit G. 1999. Detection of crosslinks with the comet assay in relationship to genotoxicity and cytotoxicity. Environ Mol Mutagen 33:167–172. doi:10.1002/(SICI)1098-2280(1999)33:2<167::AID-EM9>3.0.CO;2-D. 10217071

[32] Vizcaino MI, Crawford JM. 2015. The colibactin warhead crosslinks DNA. Nat Chem 7:411–417. doi:10.1038/nchem.2221. 25901819 PMC4499846

[33] Roos WP, Thomas AD, Kaina B. 2016. DNA damage and the balance between survival and death in cancer biology. Nat Rev Cancer 16:20–33. doi:10.1038/nrc.2015.2. 26678314

[34] Arthur JC, Gharaibeh RZ, Mühlbauer M, Perez-Chanona E, Uronis JM, McCafferty J, Fodor AA, Jobin C. 2014. Microbial genomic analysis reveals the essential role of inflammation in bacteria-induced colorectal cancer. Nat Commun 5:4724. doi:10.1038/ncomms5724. 25182170 PMC4155410

[35] Yang Y, Gharaibeh RZ, Newsome RC, Jobin C. 2020. Amending microbiota by targeting intestinal inflammation with TNF blockade attenuates development of colorectal cancer. Nat Cancer 1:723–734. doi:10.1038/s43018-020-0078-7. 33768208 PMC7990316

[36] Cevallos SA, Lee J-Y, Tiffany CR, Byndloss AJ, Johnston L, Byndloss MX, Bäumler AJ. 2019. Increased epithelial oxygenation links colitis to an expansion of tumorigenic bacteria. mBio 10:e02244-19. doi:10.1128/mBio.02244-19. 31575772 PMC6775460

[37] Dejea CM, Fathi P, Craig JM, Boleij A, Taddese R, Geis AL, Wu X, DeStefano Shields CE, Hechenbleikner EM, Huso DL, Anders RA, Giardiello FM, Wick EC, Wang H, Wu S, Pardoll DM, Housseau F, Sears CL. 2018. Patients with familial adenomatous polyposis harbor colonic biofilms containing tumorigenic bacteria. Science 359:592–597. doi:10.1126/science.aah3648. 29420293 PMC5881113

[38] Zhu W, Miyata N, Winter MG, Arenales A, Hughes ER, Spiga L, Kim J, Sifuentes-Dominguez L, Starokadomskyy P, Gopal P, Byndloss MX, Santos RL, Burstein E, Winter SE. 2019. Editing of the gut microbiota reduces carcinogenesis in mouse models of colitis-associated colorectal cancer. J Exp Med 216:2378–2393. doi:10.1084/jem.20181939. 31358565 PMC6781011

[39] Lodinová-Zádniková R, Sonnenborn U. 1997. Effect of preventive administration of a nonpathogenic *Escherichia coli* strain on the colonization of the intestine with microbial pathogens in newborn infants. Biol Neonate 71:224–232. doi:10.1159/000244421. 9129791

[40] Henker J, Laass MW, Blokhin BM, Maydannik VG, Bolbot YK, Elze M, Wolff C, Schreiner A, Schulze J. 2008. Probiotic *Escherichia coli* Nissle 1917 versus placebo for treating diarrhea of greater than 4 days duration in infants and toddlers. Pediatr Infect Dis J 27:494–499. doi:10.1097/INF.0b013e318169034c. 18469732

[41] Scaldaferri F, Gerardi V, Mangiola F, Lopetuso LR, Pizzoferrato M, Petito V, Papa A, Stojanovic J, Poscia A, Cammarota G, Gasbarrini A. 2016. Role and mechanisms of action of *Escherichia coli* Nissle 1917 in the maintenance of remission in ulcerative colitis patients: an update. World J Gastroenterol 22:5505–5511. doi:10.3748/wjg.v22.i24.5505. 27350728 PMC4917610

[42] Charbonneau MR, Isabella VM, Li N, Kurtz CB. 2020. Developing a new class of engineered live bacterial therapeutics to treat human diseases. Nat Commun 11:1738. doi:10.1038/s41467-020-15508-1. 32269218 PMC7142098

[43] Hwang IY, Koh E, Wong A, March JC, Bentley WE, Lee YS, Chang MW. 2017. Engineered probiotic *Escherichia coli* can eliminate and prevent *Pseudomonas aeruginosa* gut infection in animal models. Nat Commun 8:15028. doi:10.1038/ncomms15028. 28398304 PMC5394271

[44] Palmer JD, Piattelli E, McCormick BA, Silby MW, Brigham CJ, Bucci V. 2018. Engineered probiotic for the inhibition of *Salmonella* via tetrathionate-induced production of microcin H47. ACS Infect Dis 4:39–45. doi:10.1021/acsinfecdis.7b00114. 28918634 PMC5766358

[45] Chen Z, Guo L, Zhang Y, Walzem RL, Pendergast JS, Printz RL, Morris LC, Matafonova E, Stien X, Kang L, Coulon D, McGuinness OP, Niswender KD, Davies SS. 2014. Incorporation of therapeutically modified bacteria into gut microbiota inhibits obesity. J Clin Invest 124:3391–3406. doi:10.1172/JCI72517. 24960158 PMC4109548

[46] Isabella VM, Ha BN, Castillo MJ, Lubkowicz DJ, Rowe SE, Millet YA, Anderson CL, Li N, Fisher AB, West KA, Reeder PJ, Momin MM, Bergeron CG, Guilmain SE, Miller PF, Kurtz CB, Falb D. 2018. Development of a synthetic live bacterial therapeutic for the human metabolic disease phenylketonuria. Nat Biotechnol 36:857–864. doi:10.1038/nbt.4222. 30102294

[47] Hartwig A, Arand M, Epe B, Guth S, Jahnke G, Lampen A, Martus H-J, Monien B, Rietjens IMCM, Schmitz-Spanke S, Schriever-Schwemmer G, Steinberg P, Eisenbrand G. 2020. Mode of action-based risk assessment of genotoxic carcinogens. Arch Toxicol 94:1787–1877. doi:10.1007/s00204-020-02733-2. 32542409 PMC7303094

[48] Olier M, Marcq I, Salvador-Cartier C, Secher T, Dobrindt U, Boury M, Bacquié V, Pénary M, Gaultier E, Nougayrède J-P, Fioramonti J, Oswald E. 2012. Genotoxicity of *Escherichia coli* Nissle 1917 strain cannot be dissociated from its probiotic activity. Gut Microbes 3:501–509. doi:10.4161/gmic.21737. 22895085 PMC3495787

[49] Perez-Berezo T, Pujo J, Martin P, Le Faouder P, Galano J-M, Guy A, Knauf C, Tabet JC, Tronnet S, Barreau F, Heuillet M, Dietrich G, Bertrand-Michel J, Durand T, Oswald E, Cenac N. 2017. Identification of an analgesic lipopeptide produced by the probiotic *Escherichia coli* strain Nissle 1917. Nat Commun 8:1314. doi:10.1038/s41467-017-01403-9. 29101366 PMC5670229

[50] Tang-Fichaux M, Chagneau CV, Bossuet-Greif N, Nougayrède J-P, Oswald É, Branchu P. 2020. The polyphosphate kinase of *Escherichia coli* is required for full production of the genotoxin colibactin. mSphere 5:e01195-20. doi:10.1128/mSphere.01195-20. 33328353 PMC7771237

[51] Luo LZ, Werner KM, Gollin SM, Saunders WS. 2004. Cigarette smoke induces anaphase bridges and genomic imbalances in normal cells. Mutat Res 554:375–385. doi:10.1016/j.mrfmmm.2004.06.031. 15450433

