# Supplementary figures and images for: A Toxic Friend: Genotoxic and Mutagenic Activity of the Probiotic Strain Escherichia coli Nissle 1917
Source: mSphere. 2021 Aug 11;6(4):e00624-21. doi: 10.1128/mSphere.00624-21 (PMC8386472; doi:10.1128/mSphere.00624-21)

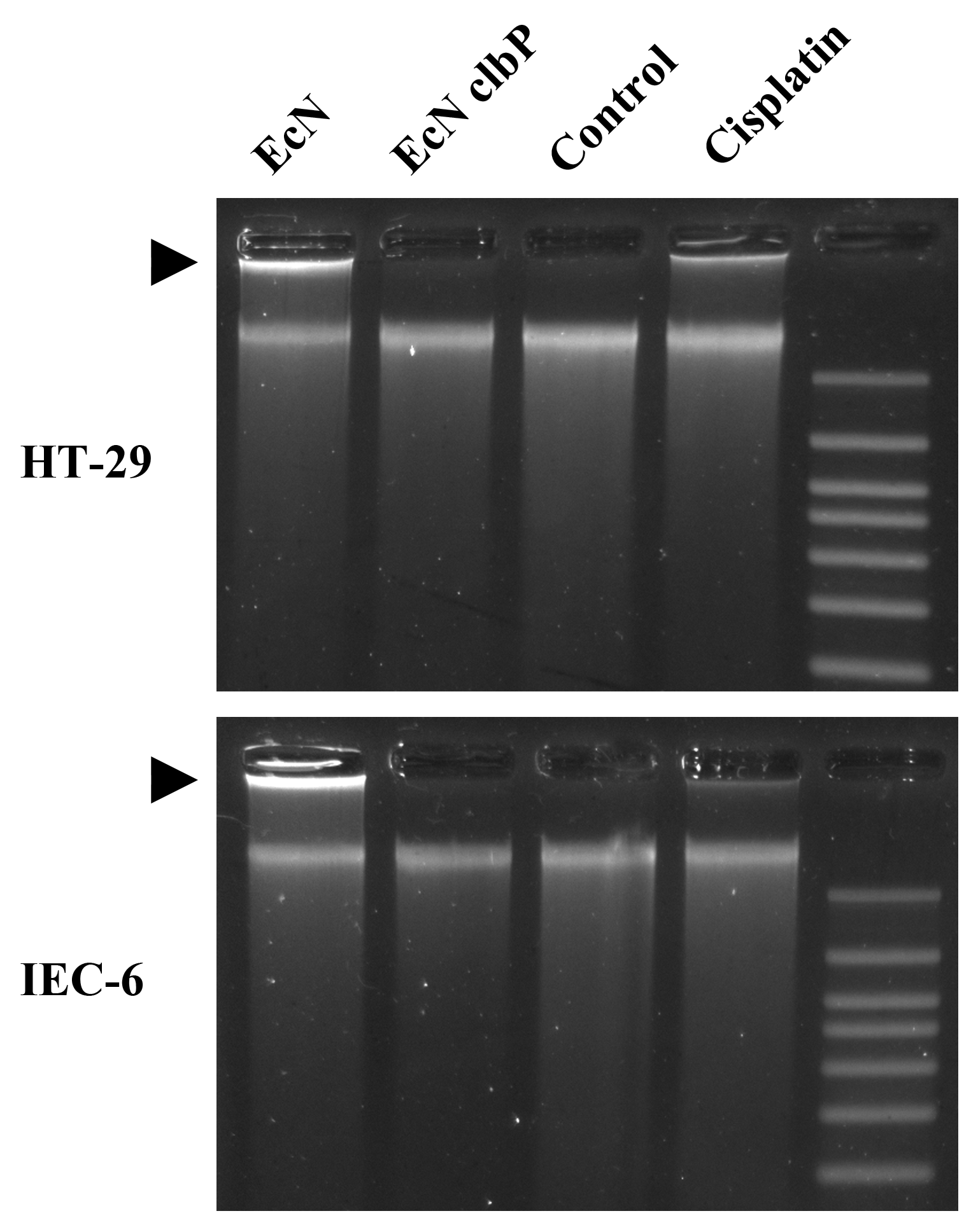

Supplement: FIG S1 [file msphere.00624-21-sf001.tif]

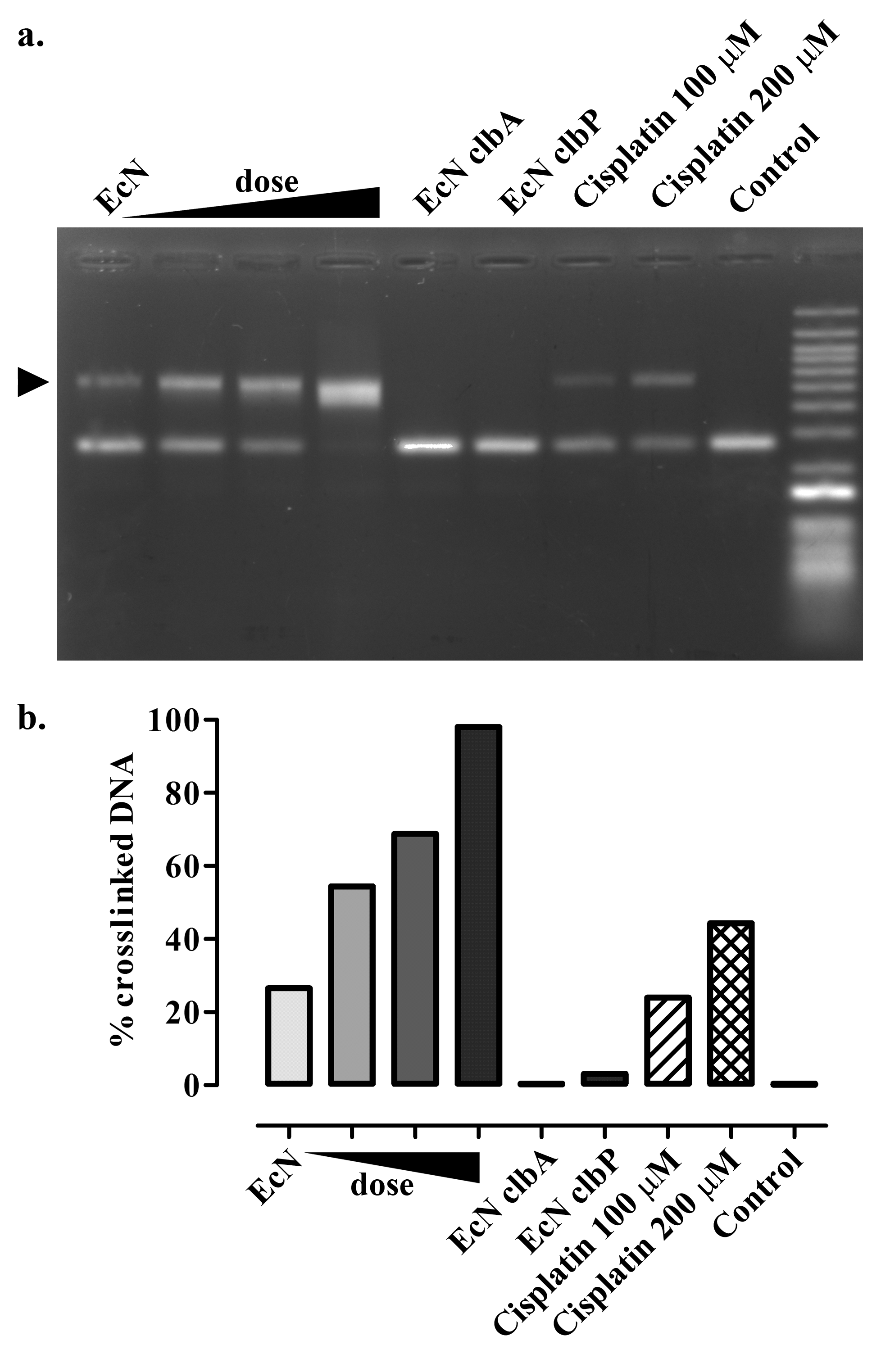

Supplement: FIG S2 [file msphere.00624-21-sf002.tif]

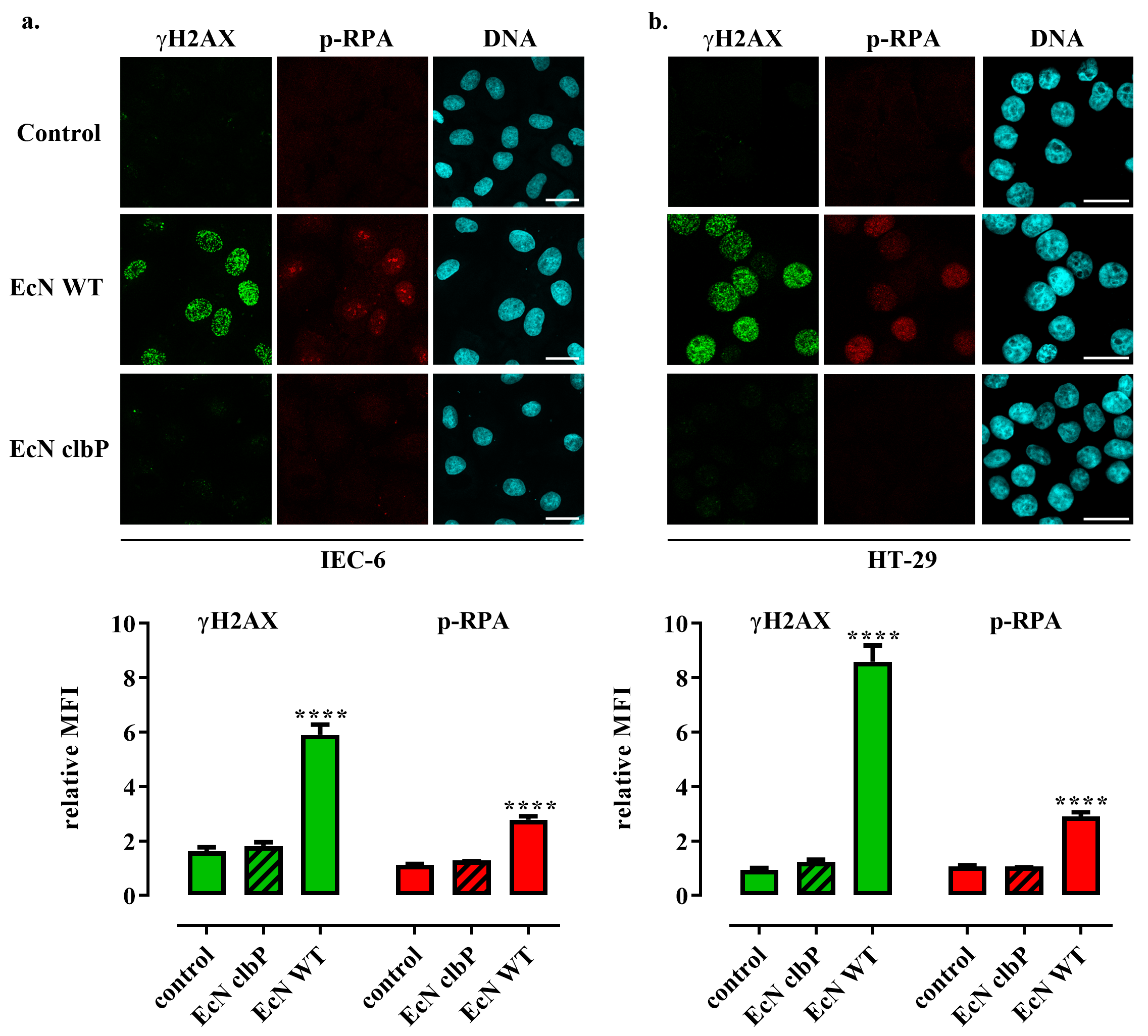

Supplement: FIG S3 [file msphere.00624-21-sf003.tif]
